# Trifluridine/tipiracil+bevacizumab (BEV) vs. fluoropyrimidine-irinotecan+BEV as second-line therapy for metastatic colorectal cancer: a randomised noninferiority trial

**DOI:** 10.1038/s41416-023-02212-2

**Published:** 2023-03-04

**Authors:** Yasutoshi Kuboki, Tetsuji Terazawa, Toshiki Masuishi, Masato Nakamura, Jun Watanabe, Hitoshi Ojima, Akitaka Makiyama, Masahito Kotaka, Hiroki Hara, Yoshinori Kagawa, Naotoshi Sugimoto, Hisato Kawakami, Atsuo Takashima, Takeshi Kajiwara, Eiji Oki, Yu Sunakawa, Soichiro Ishihara, Hiroya Taniguchi, Takako Eguchi Nakajima, Satoshi Morita, Kuniaki Shirao, Naruhito Takenaka, Daisuke Ozawa, Takayuki Yoshino

**Affiliations:** 1 Department of Experimental Therapeutics, National Cancer Center Hospital East, Kashiwa, Japan; 2 Cancer Chemotherapy Center, Osaka Medical and Pharmaceutical University, Takatsuki, Japan; 3 Department of Clinical Oncology, Aichi Cancer Center Hospital, Nagoya, Japan; 4 Aizawa Comprehensive Cancer Center, Aizawa Hospital, Matsumoto, Japan; 5 Department of Surgery, Gastroenterological Center, Yokohama City University Medical Center, Yokohama, Japan; 6 Gastrointestinal Surgery, Gunma Prefectural Cancer Center, Ota, Japan; 7 Department of Hematology/Oncology, Japan Community Healthcare Organization Kyushu Hospital, Kitakyushu, Japan; 8 Cancer Center, Gifu University Hospital, Gifu, Japan; 9 Gastrointestinal Cancer Center, Sano Hospital, Kobe, Japan; 10 Department of Gastroenterology, Saitama Cancer Center, Saitama, Japan; 11 Department of Gastrointestinal Surgery, Kansai Rosai Hospital, Amagasaki, Japan; 12 Department of Gastroenterological Surgery, Osaka General Medical Center, Osaka, Japan; 13 Department of Genetic Oncology, Osaka International Cancer Institute, Osaka, Japan; 14 Department of Medical Oncology, Kindai University Hospital, Osakasayama, Japan; 15 Department of Gastrointestinal Medical Oncology, National Cancer Center Hospital, Tokyo, Japan; 16 Department of Gastrointestinal Medical Oncology, National Hospital Organization Shikoku Cancer Center, Matsuyama, Japan; 17 Department of Surgery and Science, Graduate School of Medical Sciences, Kyushu University, Fukuoka, Japan; 18 Department of Clinical Oncology, St. Marianna University School of Medicine, Kawasaki, Japan; 19 Department of Surgical Oncology, Graduate School of Medicine, The University of Tokyo, Tokyo, Japan; 20 Department of Early Clinical Development, Kyoto University Graduate School of Medicine, Kyoto, Japan; 21 Department of Biomedical Statistics and Bioinformatics, Graduate School of Medicine, Kyoto University, Kyoto, Japan; 22 Oita University Faculty of Medicine, Oita, Japan; 23 Clinical Development and Medical Affairs Division, Taiho Pharmaceutical Co., Ltd., Tokyo, Japan; 24 Department of Gastroenterology and Gastrointestinal Oncology, National Cancer Center Hospital East, Kashiwa, Japan

**Keywords:** Oncology, Gastrointestinal cancer

## Abstract

**Background:**

This open-label, multicentre, phase II/III trial assessed the noninferiority of trifluridine/tipiracil (FTD/TPI) plus bevacizumab vs. fluoropyrimidine and irinotecan plus bevacizumab (control) as second-line treatment for metastatic colorectal cancer (mCRC).

**Methods:**

Patients were randomised (1:1) to receive FTD/TPI (35 mg/m^2^ twice daily, days 1–5 and days 8–12, 28-day cycle) plus bevacizumab (5 mg/kg, days 1 and 15) or control. The primary endpoint was overall survival (OS). The noninferiority margin of the hazard ratio (HR) was set to 1.33.

**Results:**

Overall, 397 patients were enrolled. Baseline characteristics were similar between the groups. Median OS was 14.8 vs. 18.1 months (FTD/TPI plus bevacizumab vs. control; HR 1.38; 95% confidence interval [CI] 0.99–1.93; *P*_noninferiority_ = 0.5920). In patients with a baseline sum of the diameter of target lesions of <60 mm (*n* = 216, post hoc analyses), the adjusted median OS was similar between groups (FTD/TPI plus bevacizumab vs. control, 21.4 vs. 20.7 months; HR 0.92; 95% CI 0.55–1.55). Grade ≥3 adverse events (FTD/TPI plus bevacizumab vs. control) included neutropenia (65.8% vs. 41.6%) and diarrhoea (1.5% vs. 7.1%).

**Conclusions:**

FTD/TPI plus bevacizumab did not demonstrate noninferiority to fluoropyrimidine and irinotecan plus bevacizumab as second-line treatment for mCRC.

**Clinical trial registration:**

JapicCTI-173618, jRCTs031180122.

## Background

Colorectal cancer (CRC) is the third most common cause of cancer-related mortality worldwide, with approximately 20–25% of patients presenting with metastases at initial diagnosis [1]. The combination of 5-fluorouracil (5-FU) and *l-*leucovorin (*l*-LV) with oxaliplatin (FOLFOX) or irinotecan (FOLFIRI), or both (FOLFOXIRI), along with targeted biological agents, is recommended as first- or second-line treatment options for unresectable advanced metastatic CRC (mCRC) [2, 3]. Oxaliplatin-based regimens are commonly used as first-line treatment, whereas irinotecan-based regimens are used as second-line treatment in many cases [4]. In Japan, fluoropyrimidine S-1, taken orally, is frequently used in combination with oxaliplatin or irinotecan [3].

Treatment goals for patients with mCRC are curative and focus on prolonging survival, improving tumour-related symptoms, inhibiting tumour progression, and/or maintaining the quality of life (QoL), especially during second-line treatment [5]. Irinotecan-based regimens have been used as second-line treatment for mCRC for more than a decade; however, these can cause toxic outcomes such as diarrhoea and alopecia, which may affect treatment compliance [2]. Thus, managing drug-related toxicities and maintaining the QoL are important.

Trifluridine/tipiracil (FTD/TPI) significantly improved overall survival (OS) in patients with mCRC with a history of heavily treated refractory CRC compared with placebo [6, 7]. In a preclinical study, FTD/TPI plus bevacizumab demonstrated enhanced antitumor activity against CRC xenografts when compared with either drug alone [8]. Several studies have shown promising results, including clinically relevant improvement in progression-free survival (PFS) for FTD/TPI plus bevacizumab, and evidence for using FTD/TPI plus bevacizumab as third- or later-line treatment for mCRC is growing [9–13]. FTD/TPI plus bevacizumab also showed satisfactory PFS in patients ineligible for intensive therapy and in elderly patients when used as first-line treatment [14, 15].

Compared with irinotecan-based regimens, FTD/TPI plus bevacizumab is expected to reduce the incidence of adverse events (AEs) with subjective symptoms and maintain the QoL. If FTD/TPI plus bevacizumab proves to be a feasible alternative to irinotecan-based regimens, then many patients with mCRC might have the option to avoid irinotecan and the associated AEs during second-line treatment. Therefore, the phase II/III TRiflUridine/tipiracil in Second-line sTudY (TRUSTY) aimed to demonstrate the noninferiority (in terms of OS) of FTD/TPI plus bevacizumab vs. FOLFIRI plus bevacizumab or S-1 and irinotecan plus bevacizumab as second-line treatment in Japanese patients with mCRC.

## Methods

### Study design

The study design has been reported previously [16]. Briefly, this was an open-label, multicentre, randomised, comparative, phase II/III study conducted at 65 institutions in Japan. In the phase II part of the study, safety and efficacy data were assessed in the first 50 patients of the FTD/TPI plus bevacizumab group who were subsequently evaluated for tumour response per the Response Evaluation Criteria in Solid Tumors (RECIST version 1.1) [17].

The study protocol was approved by the institutional review board or ethics committee of each institution. The study was conducted in compliance with the Declaration of Helsinki and the Clinical Trials Act [18]. This study is registered with the Japan Pharmaceutical Information Center (JapicCTI-173618) and the Japan Registry of Clinical Trials (jRCTs031180122) [16].

### Patients

The key inclusion criteria included patients aged ≥20 years with histologically confirmed mCRC who failed first-line chemotherapy with fluoropyrimidine (5-FU/*l*-LV, capecitabine, or S-1) plus oxaliplatin combined with bevacizumab, or cetuximab, or panitumumab for patients with *RAS* wildtype tumours; with an Eastern Cooperative Oncology Group performance status of 0 or 1; with evaluable lesions, as observed on imaging; and with adequate organ function [16]. Written informed consent was obtained from all patients.

### Randomisation and masking

The randomisation scheme used in the study is shown in Supplementary Fig. 1. A central electronic document management system was used to generate a random allocation sequence. For allocation, balance in the number of patients between groups at each stratification and the allocation adjustment factor level were considered. For randomisation, the minimisation method was applied after patient stratification according to *RAS* status (wildtype or mutant). Primary tumour location (left sided vs. right sided) was used as an allocation adjustment factor. Furthermore, for patients with *RAS* wildtype status, first-line treatment with a molecular-targeted drug (bevacizumab vs. anti-epidermal growth factor receptor [EGFR] antibody) was used as an allocation adjustment factor.

### Interventions

The reference treatment used in this study was identical to the standard-of-care treatment described in the Japanese guidelines for CRC treatment [3]. For patients to be assigned to the control group, the study investigators selected one of the following regimens for each patient assigned to the fluoropyrimidine and irinotecan plus bevacizumab group (control group) before enrolment: FOLFIRI plus bevacizumab (bevacizumab 5 mg/kg, irinotecan 150 mg/m^2^, and *l*-LV 200 mg/m^2^ by intravenous infusion followed by a bolus injection of 5-FU 400 mg/m^2^, all on day 1, followed by a 46-h infusion of 5-FU 2400 mg/m^2^ in a 14-day cycle); S-1 and irinotecan plus bevacizumab with a 3-week cycle (bevacizumab 7.5 mg/kg and irinotecan 150 mg/m^2^ by intravenous infusion on day 1 and oral administration of S-1 40 mg/m^2^ twice daily from days 1 to 14 in a 21-day cycle); or S-1 and irinotecan plus bevacizumab with a 4-week cycle (bevacizumab 5 mg/kg and irinotecan 100 mg/m^2^ by intravenous infusion on days 1 and 15, and oral administration of S-1 40 mg/m^2^  twice daily from days 1 to 14 in a 28-day cycle) [16]. Patients assigned to the FTD/TPI plus bevacizumab group received bevacizumab 5 mg/kg by intravenous infusion on days 1 and 15 and oral administration of FTD/TPI 35 mg/m^2^ twice daily on days 1–5 and 8–12 in a 28-day cycle. Diagnostic imaging was performed every 8 weeks (±2 weeks) until disease progression, and tumour responses were assessed by the site investigators per RECIST version 1.1 [17].

### Outcomes

The primary endpoint in the phase II part of the study was the disease control rate (DCR), which was the proportion of complete or partial responses or stable disease for more than 6 weeks from the initiation of study treatment. The primary endpoint in phase III was OS, defined as the period from the date of enrolment to the date of death from any cause [16]. Secondary endpoints were QoL, PFS (period from the date of enrolment to the earliest date of disease progression or death due to any cause, whichever occurs first), response rate (RR; proportion of patients with complete or partial response), DCR, safety, time to treatment failure (TTF; period from the date of enrolment to the earliest date of disease progression, withdrawal of study treatment for any reason, or death due to any cause, whichever occurs first), and time to post-study treatment failure (TTF2; defined as the period from the date of enrolment to the date of discontinuation of third-line treatment [post-study treatment]. If no third-line treatment was administered, then TTF2 was defined as the period from the date of enrolment to the date of discontinuation of second-line treatment [protocol treatment]). AEs were graded according to the National Cancer Institute Common Terminology Criteria for Adverse Events version 4.03 [19]. The European Organisation for Research and Treatment of Cancer Quality of Life Questionnaire Core 30 (EORTC QLQ-C30) [20] and the 5-level version of EuroQoL (EQ-5D-5L) [21] were used to assess patients’ QoL before treatment initiation and every 4 weeks (±1 week) up to week 24, and every 24 weeks (±2 weeks) thereafter until discontinuation of study treatment for EORTC QLQ-C30 or until death for EQ-5D-5L.

### Statistical analysis

Based on the results from previous studies [22, 23] and improved treatment options for patients with mCRC, the median OS was assumed to be 19 months in both groups. Using the effect retention method [24], the noninferiority margin of the HR was set as 1.33, which retains approximately 60% of the effect of the control group on best supportive care (6.5 months) [25]. With a one-sided alpha significance level of 2.5%, power of 80%, 24-month enrolment period, and 30-month follow-up period after the last patient enrolment, 499 (387 events) patients were required. Assuming that approximately 5% of patients were excluded from the full analysis set (FAS), the target number of patients was set at 524.

Based on recommendations from an independent data monitoring committee (IDMC) when evaluating the phase II part of the study, an interim analysis for futility, wherein the primary endpoint was OS in the phase III study, was planned and represented as Cox proportional hazards and Bayesian prediction probability. The IDMC comprehensively evaluated whether the study was to be continued.

Time-dependent events were estimated using the Kaplan–Meier method. HRs and their confidence intervals (CIs) were calculated using the stratified Cox proportional hazards model. The stratified factors were as follows: patients with a *RAS* mutation were stratified by primary tumour location (left sided vs. right sided) only, whereas those with *RAS* wildtype were stratified by primary tumour location and first-line treatment with a molecular-targeted drug (bevacizumab vs. anti-EGFR). If the upper limit of the 95% CI of the HR did not exceed 1.33, a noninferiority margin of 1.25 and 1.00 (superiority) was considered [16]. Safety data were summarised using descriptive statistics. The QoL analysis estimated the time to a clinically relevant difference in the QoL scores. Clinically relevant difference was defined as 0.05 relative to baseline in the EQ-5D-5L utility index score and 10 relative to baseline in the EORTC QLQ-C30. Post hoc analyses were performed for the subgroups: intent-to-use 5-FU or S-1 and the baseline sum of the diameter of target lesions (STL) for which an interaction was found, and CIs were calculated as described above. Survival curves for OS were investigated by direct survival estimation adjusted for stratification factors because some baseline characteristics were unbalanced.

The analysis groups comprised FAS (all randomised patients except those with serious protocol violations or who withdrew consent to participate), safety analysis set (all patients in FAS who received ≥1 dose of the study drug in their assigned treatment group), and QoL analysis set (all patients in FAS from whom QoL questionnaires were collected before the initiation of study treatment and ≥1 timepoint after treatment initiation). All statistical analyses were performed using SAS^®^ software, version 9.4 (SAS Institute, Cary, NC, USA).

## Results

**Fig. 1:**
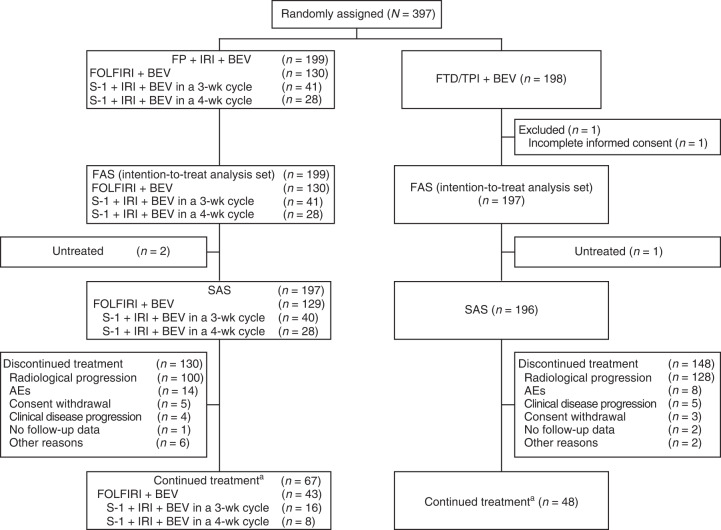
CONSORT diagram. AE adverse event, BEV bevacizumab, FAS full analysis set, FOLFIRI 5-fluorouracil, leucovorin, and irinotecan, FP fluoropyrimidine, FTD/TPI trifluridine/tipiracil, IRI irinotecan, SAS safety analysis set, wk week. ^a^As of data cutoff (16 July 2020).

Between 2 October 2017, and 16 July 2020, 397 patients were enrolled from 65 institutions: 197 patients in the FTD/TPI plus bevacizumab group (1 patient was excluded owing to incomplete informed consent), and 199 patients in the control group were included in the FAS (Fig. 1). The IDMC reviewed the results of the phase II part of the study and provided approval for proceeding to phase III. Based on the results of the interim analysis for futility, performed with combined data from phases II and III, the IDMC recommended termination of the study in July 2020 because of a low possibility for noninferiority even if the study had been completed, which the steering committee accepted.

The baseline characteristics of patients were similar between the two groups (Table 1). The relative dose intensity between the FTD/TPI plus bevacizumab and the control groups is shown in Supplementary Table 1. The median treatment duration was 3.3 months (range, 0–23.5) in the FTD/TPI plus bevacizumab group and 4.2 months (range, 0–24.4) in the control group.

**Table 1:** Baseline characteristics of patients in the FTD/TPI plus BEV group and the control group in the full analysis set.

Characteristics	FP plus IRI plus BEV (*n* = 199)	FTD/TPI plus BEV (*n* = 197)
Sex		
Male	99 (49.7)	94 (47.7)
Female	100 (50.3)	103 (52.3)
Age, years	68.0 (32–82)	67.0 (25–84)
<65	75 (37.7)	80 (40.6)
≥65	124 (62.3)	117 (59.4)
ECOG performance status		
0	124 (62.3)	120 (60.9)
1	75 (37.7)	77 (39.1)
*RAS* status		
Wildtype	79 (39.7)	79 (40.1)
Mutant	120 (60.3)	118 (59.9)
Primary tumour location^a^		
Left side	149 (74.9)	150 (76.1)
Right side	50 (25.1)	47 (23.9)
No. of metastatic lesions		
0 or 1	82 (41.2)	70 (35.5)
≥2	117 (58.8)	127 (64.5)
PFS of first-line treatment		
≥9 months	131 (65.8)	130 (66.0)
<9 months	68 (34.2)	67 (34.0)
Biologics used in first-line treatment		
Anti-EGFR antibody	35 (17.6)	37 (18.8)
BEV	164 (82.4)	160 (81.2)
Intent-to-use 5-FU or S-1^b^		
FOLFIRI plus BEV	130 (65.3)	125 (63.5)
S-1 and IRI plus BEV	69 (34.7)	72 (36.5)

Data are presented as *n* (%) or median (range).

*5-FU* 5-fluorouracil, *BEV* bevacizumab, *ECOG* Eastern Cooperative Oncology Group, *EGFR* epidermal growth factor receptor, *FOLFIRI* 5-FU, leucovorin, and irinotecan, *FP* fluoropyrimidine, *FTD/TPI* trifluridine/tipiracil, *IRI* irinotecan, *PFS* progression-free survival, *RAS* rat sarcoma virus.

^a^Tumours located in the caecum, ascending colon, and transverse colon were considered right sided; tumours within the splenic flexure and beyond were considered left sided.

^b^Before randomisation, the use of either 5-FU or S-1 was declared by each investigator when allocated to the FP plus IRI plus BEV group.

**Fig. 2:**
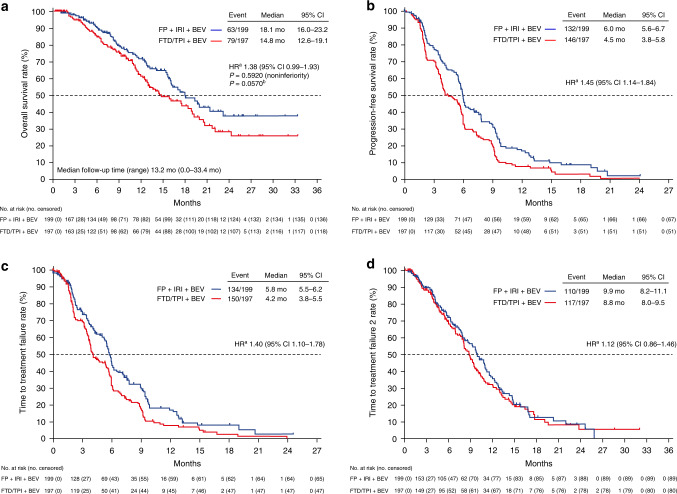
Kaplan–Meier curves. **a** overall survival, **b** progression-free survival, **c** time to treatment failure, and **d** time to treatment failure 2. BEV bevacizumab, CI confidence interval, FP fluoropyrimidine, FTD/TPI trifluridine/tipiracil, HR hazard ratio, IRI irinotecan, mo months. ^a^Adjusted based on stratification factors. ^b^Ad hoc unplanned two-sided superiority test.

The median duration of follow-up was 13.2 months (range, 0.0–33.4) as of 16 July 2020 (data cutoff). The median OS was 14.8 months (95% CI 12.6–19.1) in the FTD/TPI plus bevacizumab group and 18.1 months (95% CI 16.0–23.2) in the control group (HR 1.38; 95% CI 0.99–1.93; upper limit of the HR above the noninferiority margin of 1.33, *P* = 0.592 for noninferiority); the noninferiority of FTD/TPI plus bevacizumab was not demonstrated (Fig. 2a). The median PFS, TTF, and TTF2 were, respectively, 4.5, 4.2, and 8.8 months in the FTD/TPI plus bevacizumab group and 6.0, 5.8, and 9.9 months in the control group (Fig. 2b–d). The RR and DCR were 3.8% and 61.2% in the FTD/TPI plus bevacizumab group and 7.1% and 71.7% in the control group, respectively (Supplementary Table 2).

**Fig. 3:**
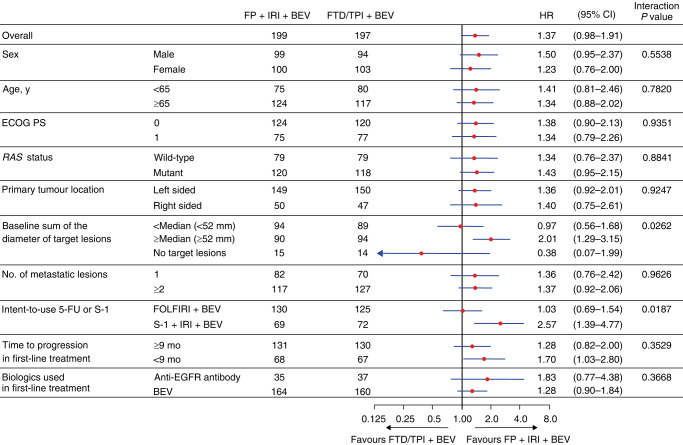
Subgroup baseline analyses of overall survival. 5-FU 5-fluorouracil, BEV bevacizumab, CI confidence interval, ECOG PS Eastern Cooperative Oncology Group performance status, EGFR epidermal growth factor receptor, FOLFIRI 5-FU, leucovorin, and irinotecan, FP fluoropyrimidine, FTD/TPI trifluridine/tipiracil, HR hazard ratio, IRI irinotecan, *RAS* rat sarcoma virus, y years.

In the subgroup analyses of OS, significant interactions were noted between the assigned regimen and the intent-to-use 5-FU or S-1 (*P* = 0.0187) and STL (median 52 mm; *P* = 0.0262; Fig. 3).

**Fig. 4:**
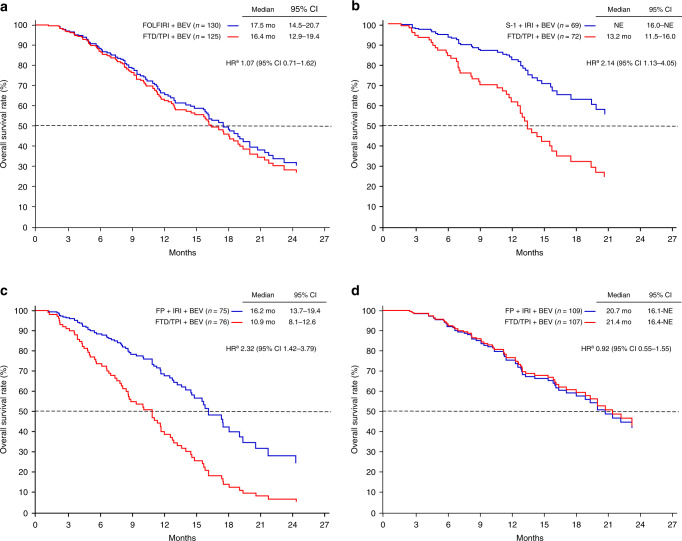
Adjusted overall survival curves. **a** intent-to-use 5-FU, **b** intent-to-use S-1, **c** baseline sum of the diameter of target lesions ≥60 mm, and **d** baseline sum of the diameter of target lesions <60 mm. 5-FU 5-fluorouracil, BEV bevacizumab, CI confidence interval, FOLFIRI 5-FU, leucovorin, and irinotecan, FP fluoropyrimidine, FTD/TPI trifluridine/tipiracil, HR hazard ratio, IRI irinotecan, NE not estimable. ^a^Adjusted based on stratification factors.

As for patient characteristics according to the intent-to-use 5-FU or S-1, there was a difference in *RAS* status between the two treatment groups, which affected the use of anti-EGFR treatment as a first-line treatment (Supplementary Table 3). In post hoc analyses, the adjusted median OS among patients with the intent-to-use 5-FU was 16.4 months in the FTD/TPI plus bevacizumab group and 17.5 months in the control group. The adjusted median OS among patients with the intent-to-use S-1 was 13.2 months in the FTD/TPI plus bevacizumab group. OS was not estimable in the S-1 and irinotecan plus bevacizumab groups (Fig. 4a, b).

Because there was an interaction at STL (median 52 mm), we examined the optimal cutoff for STL, and it was regarded as 60 mm, which had the most significant difference level for OS (Supplementary Fig. 2 and Supplementary Table 4). There was no clinical difference in patient characteristics between the FTD/TPI plus bevacizumab and control groups according to STL (Supplementary Table 5). In post hoc analyses, the adjusted median OS in patients with STL ≥60 mm was 10.9 months in the FTD/TPI plus bevacizumab group and 16.2 months in the control group (HR 2.32; 95% CI 1.42–3.79). Conversely, the adjusted median OS in patients with STL <60 mm was 21.4 months in the FTD/TPI plus bevacizumab group and 20.7 months in the control group (HR 0.92; 95% CI 0.55–1.55; Fig. 4c, d).

The most common grade ≥3 AEs in the FTD/TPI plus bevacizumab group and the control group were neutropenia (129 [65.8%] vs. 82 [41.6%]), leukopenia (49 [25.0%] vs. 18 [9.1%]), anaemia (12 [6.1%] vs. 6 [3.0%]), diarrhoea (3 [1.5%] vs. 14 [7.1%]), and anorexia (5 [2.6%] vs. 12 [6.1%]). Alopecia (all grades) was reported in 7 (3.6%) patients in the FTD/TPI plus bevacizumab group and 49 (24.9%) patients in the control group (Table 2). Grade ≥3 febrile neutropenia was reported in 4 (2.0%) patients in the FTD/TPI plus bevacizumab group and 5 (2.5%) in the control group. A total of 17 (8.7%) patients in the FTD/TPI plus bevacizumab group and 19 (9.6%) patients in the control group received granulocyte colony-stimulating factor. Serious adverse drug reactions (ADRs) were observed in 10 (5.1%) patients in the FTD/TPI plus bevacizumab group and 28 (14.2%) patients in the control group. One treatment-related death occurred in the FTD/TPI plus bevacizumab group owing to cerebral infarction.

**Table 2:** Commonly reported adverse events in the safety analysis set.

Adverse events	FTD/TPI plus BEV (*n* = 196)		FP plus IRI plus BEV (*n* = 197)	
	All	Grade ≥3	All	Grade ≥3
All events	188 (95.9)	152 (77.6)	188 (95.4)	131 (66.5)
Haematological				
Leukopenia	85 (43.4)	49 (25.0)	36 (18.3)	18 (9.1)
Neutropenia	154 (78.6)	129 (65.8)	124 (62.9)	82 (41.6)
Thrombocytopenia	37 (18.9)	9 (4.6)	21 (10.7)	2 (1.0)
Anaemia	44 (22.4)	12 (6.1)	20 (10.2)	6 (3.0)
Nonhaematological				
Febrile neutropenia	4 (2.0)	4 (2.0)	5 (2.5)	5 (2.5)
Stomatitis	29 (14.8)	1 (0.5)	48 (24.4)	3 (1.5)
Nausea	59 (30.1)	2 (1.0)	61 (31)	4 (2.0)
Vomiting	20 (10.2)	0	20 (10.2)	2 (1.0)
Diarrhoea	63 (32.1)	3 (1.5)	81 (41.1)	14 (7.1)
Anorexia	86 (43.9)	5 (2.6)	70 (35.5)	12 (6.1)
Fatigue	42 (21.4)	4 (2.0)	38 (19.3)	6 (3.0)
Alopecia	7 (3.6)	NA	49 (24.9)	NA

Data are presented as *n* (%).

*BEV* bevacizumab, *FP* fluoropyrimidine, *FTD/TPI* trifluridine/tipiracil, *IRI* irinotecan, *NA* not available.

Overall, 118/149 (79.2%) patients in the FTD/TPI plus bevacizumab group and 104/132 (78.8%) patients in the control group received third-line treatment (excluding patients who continued study treatment until data cutoff). As a third-line drug, 104/118 (88.1%) patients in the FTD/TPI plus bevacizumab group and 24/104 (23.1%) patients in the control group received irinotecan; 44/104 (42.3%) patients in the control group received FTD/TPI (33/44 patients were treated with bevacizumab; Supplementary Table 6).

Overall, 173 (86.9%) patients in the FTD/TPI plus bevacizumab group and 181 (91.4%) patients in the control group were included in the QoL analysis set. There were no differences in the time to deterioration of the EQ-5D-5L utility index score between the groups (Supplementary Fig. 3). In the EORTC QLQ-C30, the FTD/TPI plus bevacizumab group showed better results for nausea and vomiting symptoms and pain symptoms than the control group (Supplementary Fig. 3).

## Discussion

This is the first phase II/III trial to verify the efficacy of FTD/TPI plus bevacizumab as a second-line treatment for patients with mCRC. FTD/TPI plus bevacizumab did not demonstrate noninferiority to fluoropyrimidine and irinotecan plus bevacizumab in terms of OS.

PFS and DCR in the FTD/TPI plus bevacizumab group were similar to those reported in the third- or late-line setting [9–13]. FTD/TPI plus bevacizumab showed promising results in the first-line setting [14, 15]; thus, we expected better results with FTD/TPI plus bevacizumab as a second-line treatment without irinotecan. FTD/TPI plus bevacizumab was expected to reduce the incidence of adverse events with subjective symptoms compared with fluoropyrimidine and irinotecan plus bevacizumab. We assumed that irinotecan would be used as a third-line treatment in the FTD/TPI plus bevacizumab group. It was expected to maintain QoL over the entire treatment duration by reducing the toxicity of second-line treatment, which generally has a longer treatment duration than third-line treatment. In addition, it was assumed that the use of irinotecan in the second- or third-line treatment would result in equivalent OS in both arms; however, this was not true. The importance of the efficacy of second-line treatment for mCRC was thus reaffirmed. Differences in PFS could not be recovered by TTF2. The TTF2 obtained from this study can be adequately evaluated as a study of the second-line setting because post-study treatment was well conducted in both groups.

The study was planned assuming that FOLFIRI plus bevacizumab and S-1 and irinotecan plus bevacizumab had similar efficacy; however, in the post hoc analyses, median OS in the S-1 and irinotecan plus bevacizumab treatment tended to be longer than that in the FOLFIRI plus bevacizumab treatment. Some trials have shown that oral fluoropyrimidine plus irinotecan might be more effective than intravenous 5-FU plus irinotecan or oxaliplatin [22, 26, 27]. Careful interpretation is required because of the differences in baseline characteristics (especially *RAS* status) between the FTD/TPI plus bevacizumab group and the control group.

Although the results of the STL are based on post hoc analyses, OS was similar between the FTD/TPI plus bevacizumab group and the control group in patients with low tumour burden (STL <60 mm). The baseline characteristics in patients with both high and low tumour burden were balanced between the treatment groups. The SOLSTICE phase III study was conducted in patients with mCRC who were ineligible to receive standard full-dose doublet regimens with oxaliplatin or irinotecan and included approximately 13% of patients with low tumour burden [28]. FTD/TPI plus bevacizumab did not show significant superiority in terms of PFS when compared with capecitabine plus bevacizumab; the median PFS was 9.4 months vs. 9.3 months (HR 0.87; 95% CI 0.75–1.02; *P* = 0.0464 [<0.021 to be significant]) [28]. However, FTD/TPI plus bevacizumab was clinically satisfactory in patients with mCRC who were ineligible for intensive chemotherapy. FTD/TPI plus bevacizumab might be the preferred treatment for patients with low tumour burden in terms of PFS (HR 0.68) [28]. In the TASCO1 study, 20% of patients were ineligible to receive intensive treatment because of the low tumour burden [14]. In a post hoc exploratory analysis of the randomised, double-blind, phase 3 study of FTD/TPI plus best supportive care (BSC) versus placebo plus BSC in patients with mCRC refractory to standard chemotherapies (RECOURSE) trial, low tumour burden was shown to be a factor of good prognosis in late-line mCRC [29]. Furthermore, in an exploratory analysis based on an 18-month registry of the patients with mCRC treated with FTD/TPI that evaluated the impact of patient stratification by prognosis group, low tumour burden was one of the factors that influenced sustained response to FTD/TPI, with a positive impact on median OS and median PFS [30]. Such patients may benefit from treatment with FTD/TPI plus bevacizumab. This regimen might also be effective in patients with slow-growing tumours [31]. However, as there is no consensus on the definition of low tumour burden, it is important to consider the tumour size, number of metastatic lesions, and metastasis site when administering treatment.

The safety profile of FTD/TPI plus bevacizumab is consistent with that reported in other studies [11, 12, 14]. Haematological toxicities occurred more frequently in the FTD/TPI plus bevacizumab group, whereas nonhaematological toxicities occurred more frequently in the control group. Serious ADRs occurred more frequently in the control group. Thus, FTD/TPI plus bevacizumab will provide an alternative to irinotecan for oncologists to treat patients who cannot tolerate irinotecan-containing regimens due to nonhaematological toxicities. Patients in the FTD/TPI plus bevacizumab group tended to have a better QoL than those in the control group.

Although this study did not show noninferiority of FTD/TPI plus bevacizumab to the control group, the FTD/TPI plus bevacizumab regimen remains a promising treatment because the findings suggest its use as third- or later-line treatment. In a randomised, phase II, open-label study of patients receiving therapy for refractory mCRC, treatment with FTD/TPI plus bevacizumab vs. FTD/TPI monotherapy was associated with a significant improvement in PFS (4.6 vs. 2.6 months; HR 0.45; *P* = 0.0015) [11]. Based on this result, a confirmatory phase III study (SUNLIGHT study) of the FTD/TPI plus bevacizumab regimen as a third-line treatment for patients with mCRC is ongoing [32].

Limitations of the study include the short follow-up period owing to early study termination. A total of 100 patients (39.4%) had a follow-up period of ≤6 months at the time of study discontinuation. In addition, the optimal cutoff for STL was regarded as 60 mm, which had the most significant difference level for OS; however, this cutoff point was not validated. Further analysis is needed to determine whether this cutoff for low tumour burden is appropriate in other studies.

In conclusion, our results show that fluoropyrimidine and irinotecan plus bevacizumab remain the standard second-line treatment for mCRC. For patients with low tumour burden, further investigations are warranted to explore more appropriate second-line treatment.

## Supplementary information

Supplementary material

## Data Availability

The datasets used and/or analysed during the current study are available from the corresponding author upon reasonable request.
