# Supplementary material for: Trifluridine/tipiracil+bevacizumab (BEV) vs. fluoropyrimidine-irinotecan+BEV as second-line therapy for metastatic colorectal cancer: a randomised noninferiority trial
Source: Br J Cancer. 2023 Mar 4;128(10):1897–905. doi: 10.1038/s41416-023-02212-2 (PMC10147634; doi:10.1038/s41416-023-02212-2)
Supplement: Supplementary file 1 — Supplementary material [file 41416_2023_2212_MOESM1_ESM.docx]

**SUPPLEMENTARY MATERIAL**

**Supplementary Table 1. Relative dose intensity in patients included in the safety analysis.**

|  | **FTD/TPI plus BEV**  **(*n*=196), mean** | **FP plus IRI plus BEV**  **(*n* = 197), mean** | | |
| --- | --- | --- | --- | --- |
|  |  | **Total** | **FOLFIRI plus BEV (*n* = 129)** | **S-1 plus IRI plus BEV (*n* = 68)** |
| 5-FU continuous infusion | NA | NA | 75.3 | NA |
| S-1 | NA | NA | NA | 70.0 |
| IRI | NA | 76.5 | 76.8 | 75.9 |
| FTD/TPI | 79.7 | NA | NA | NA |
| BEV | 77.9 | 79.1 | 78.9 | 86.1 |

5-FU, 5-fluorouracil; BEV, bevacizumab; FOLFIRI, 5-FU, leucovorin, and irinotecan; FP, fluoropyrimidine; FTD/TPI, trifluridine/tipiracil; IRI, irinotecan; NA, not available.

**Supplementary Table 2. Best overall response in tumor response–evaluable patients.**

|  | **FTD/TPI plus BEV (*n* = 183)^a^** | **FP plus IRI plus BEV (*n* = 184)^a^** | ***P* value** |
| --- | --- | --- | --- |
| Response |  |  |  |
| CR | 0 | 0 |  |
| PR | 7 (3.8) | 13 (7.1) |  |
| SD | 105 (57.4) | 119 (64.7) |  |
| PD | 45 (24.6) | 25 (13.6) |  |
| NE | 26 (14.2) | 27 (14.7) |  |
| Response rate  (CR or PR) | 7 (3.8) | 13 (7.1) | 0.2498 |
| 95% CI, % | 1.6-7.7 | 3.8-11.8 |  |
| Disease control rate  (CR or PR or SD) | 112 (61.2) | 132 (71.7) | 0.0359 |
| 95% CI, % | 53.7-68.3 | 64.6-78.1 |  |

Data are presented as *n* (%).

^a^No. of patients with measurable lesions according to RECIST, version 1.1, based on investigator assessment.

BEV, bevacizumab; CI, confidence interval; CR, complete response; FP, fluoropyrimidine; FTD/TPI, trifluridine/tipiracil; IRI, irinotecan; NE, not estimable; PD, progressive disease; PR, partial response; RECIST, Response Evaluation Criteria in Solid Tumors; SD, stable disease.

**Supplementary Table 3. Baseline characteristics of patients according to intent-to-use 5-FU or S-1 in the full analysis set.**

|  | **Intent-to-use 5-FU** | | **Intent-to-use S-1** | |
| --- | --- | --- | --- | --- |
| **Baseline characteristics** | **FTD/TPI plus BEV (*n* = 125)** | **FP plus IRI plus BEV (*n* = 130)** | **FTD/TPI plus BEV (*n* = 72)** | **FP plus IRI plus BEV (*n* = 69)** |
| Sex |  | | | |
| Male | 61 (48.8) | 62 (47.7) | 33 (45.8) | 37 (53.6) |
| Female | 64 (51.2) | 68 (52.3) | 39 (54.2) | 32 (46.4) |
| Age, y |  | | | |
| Median (range) | 66.0 (37-82) | 68.0 (32-82) | 68.0 (25-84) | 67.0 (40-81) |
| <65 | 53 (42.4) | 47 (36.2) | 27 (37.5) | 28 (40.6) |
| ≥65 | 72 (57.6) | 83 (63.8) | 45 (62.5) | 41 (59.4) |
| ECOG performance status |  | | | |
| 0 | 74 (59.2) | 76 (58.5) | 46 (63.9) | 48 (69.6) |
| 1 | 51 (40.8) | 54 (41.5) | 26 (36.1) | 21 (30.4) |
| *RAS* status |  | | | |
| Wild type | 64 (51.2) | 51 (39.2) | 13 (18.1) | 27 (39.1) |
| Mutant | 61 (48.8) | 79 (60.8) | 59 (81.9) | 42 (60.9) |
| Primary tumor location |  | | | |
| Left side | 97 (77.6) | 100 (76.9) | 53 (73.6) | 49 (71.0) |
| Right side | 28 (22.4) | 30 (23.1) | 19 (26.4) | 20 (29.0) |
| No. of metastatic lesions |  | | | |
| 0 or 1 | 44 (35.2) | 54 (41.5) | 26 (36.1) | 28 (40.6) |
| ≥2 | 81 (64.8) | 76 (58.5) | 46 (63.9) | 41 (59.4) |
| PFS of first-line treatment |  | | | |
| ≥9 mo | 81 (64.8) | 81 (62.3) | 49 (68.1) | 50 (72.5) |
| <9 mo | 44 (35.2) | 49 (37.7) | 23 (31.9) | 19 (27.5) |
| Biologics used in first-line treatment |  | | | |
| Anti-EGFR antibody | 32 (25.6) | 27 (20.8) | 5 (6.9) | 8 (11.6) |
| BEV | 93 (74.4) | 103 (79.2) | 67 (93.1) | 61 (88.4) |

Data are presented as *n* (%) or median (range).

5-FU, 5-fluorouracil; BEV, bevacizumab; ECOG, Eastern Cooperative Oncology Group; EGFR, epidermal growth factor receptor; FP, fluoropyrimidine; FTD/TPI, trifluridine/tipiracil; IRI, irinotecan; mo, months; PFS, progression-free survival; *RAS*, rat sarcoma virus; y, years.

**Supplementary Table 4. Overall survival by the baseline sum of the diameter of target lesions.**

|  | **FTD/TPI plus BEV** | | **FP plus IRI plus BEV** | | **HR (95% CI)** | **Interaction *P* value** |
| --- | --- | --- | --- | --- | --- | --- |
| **Baseline STL, mm** | ***N*** | **MST, mo** | ***N*** | **MST, mo** |  |  |
| <Median | 89 | 20.7 | 94 | 21.4 | 0.97 (0.56-1.68) | 0.0387 |
| ≥Median | 94 | 11.5 | 90 | 17.5 | 2.01 (1.29-3.15) |  |
| <20 | 21 | 20.0 | 22 | NA | 1.74 (0.49-6.23) | 0.7303 |
| ≥20 | 162 | 14.0 | 162 | 17.4 | 1.44 (1.01-2.06) |  |
| <25 | 30 | 22.2 | 36 | 36.0 | 1.03 (0.37-2.85) | 0.5616 |
| ≥25 | 153 | 13.0 | 148 | 17.5 | 1.50 (1.04-2.15) |  |
| <30 | 45 | NA | 50 | NA | 0.89 (0.38-2.11) | 0.2065 |
| ≥30 | 138 | 11.7 | 134 | 17.5 | 1.65 (1.13-2.40) |  |
| <35 | 53 | 23.2 | 62 | NA | 0.95 (0.44-2.06) | 0.214 |
| ≥35 | 130 | 11.7 | 122 | 17.5 | 1.63 (1.11-2.39) |  |
| <40 | 61 | 23.2 | 74 | NA | 0.99 (0.50-1.98) | 0.1937 |
| ≥40 | 122 | 11.7 | 110 | 17.5 | 1.64 (1.10-2.44) |  |
| <45 | 78 | 21.4 | 81 | NA | 1.20 (0.66-2.21) | 0.349 |
| ≥45 | 105 | 11.7 | 103 | 17.4 | 1.67 (1.11-2.53) |  |
| <50 | 86 | 20.0 | 88 | 23.2 | 1.12 (0.64-1.98) | 0.1677 |
| ≥50 | 97 | 11.5 | 96 | 17.5 | 1.83 (1.19-2.81) |  |
| <55 | 98 | 20.0 | 97 | 21.4 | 1.00 (0.59-1.70) | 0.0342 |
| ≥55 | 85 | 11.1 | 87 | 17.4 | 2.1 (1.33-3.32) |  |
| <60 | 107 | 21.4 | 109 | 20.7 | 0.94 (0.57-1.56) | 0.012 |
| ≥60 | 76 | 10.9 | 75 | 16.2 | 2.27 (1.41-3.66) |  |
| <65 | 114 | 20.7 | 113 | 21.4 | 1.05 (0.64-1.71) | 0.0405 |
| ≥65 | 69 | 10.9 | 71 | 16.2 | 2.13 (1.32-3.46) |  |
| <70 | 120 | 20.0 | 119 | 22.2 | 1.13 (0.70-1.82) | 0.0505 |
| ≥70 | 63 | 10.9 | 65 | 15.7 | 2.19 (1.33-3.59) |  |
| <75 | 124 | 19.3 | 125 | 21.4 | 1.17 (0.73-1.85) | 0.1033 |
| ≥75 | 59 | 10.1 | 59 | 15.6 | 2.01 (1.20-3.38) |  |
| <80 | 130 | 18.9 | 127 | 22.2 | 1.31 (0.84-2.06) | 0.3155 |
| ≥80 | 53 | 10.1 | 57 | 14.6 | 1.83 (1.08-3.11) |  |
| <85 | 133 | 18.0 | 134 | 21.4 | 1.35 (0.88-2.08) | 0.4904 |
| ≥85 | 50 | 9.4 | 50 | 14.5 | 1.67 (0.95-2.94) |  |
| <90 | 138 | 17.1 | 140 | 20.7 | 1.39 (0.92-2.12) | 0.757 |
| ≥90 | 45 | 9.4 | 44 | 13.7 | 1.55 (0.86-2.80) |  |
| <95 | 145 | 16.2 | 143 | 20.7 | 1.47 (0.98-2.21) | 0.9346 |
| ≥95 | 38 | 8.7 | 41 | 12.8 | 1.49 (0.80-2.79) |  |
| <100 | 149 | 16.2 | 144 | 21.4 | 1.51 (1.01-2.26) | 0.9315 |
| ≥100 | 34 | 8.7 | 40 | 12.6 | 1.44 (0.76-2.72) |  |
| <105 | 151 | 16.1 | 149 | 21.4 | 1.54 (1.03-2.31) | 0.7571 |
| ≥105 | 32 | 8.7 | 35 | 11.7 | 1.35 (0.71-2.57) |  |
| <110 | 155 | 16.1 | 149 | 21.4 | 1.54 (1.03-2.31) | 0.9076 |
| ≥110 | 28 | 8.7 | 35 | 11.7 | 1.45 (0.75-2.77) |  |
| <115 | 158 | 16.0 | 154 | 20.7 | 1.47 (1.00-2.18) | 0.7011 |
| ≥115 | 25 | 8.7 | 30 | 12.8 | 1.67 (0.83-3.38) |  |
| <120 | 161 | 15.9 | 161 | 20.0 | 1.49 (1.02-2.18) | 0.7207 |
| ≥120 | 22 | 9.4 | 23 | 9.4 | 1.24 (0.57-2.72) |  |

BEV, bevacizumab; CI, confidence interval; FP, fluoropyrimidine; FTD/TPI, trifluridine/tipiracil; HR, hazard ratio; IRI, irinotecan; mo, months; MST, median survival time; NA, not available; STL, sum of the diameter of target lesions.

**Supplementary Table 5. Baseline characteristics of patients according to the baseline sum of the diameter of target lesions in the full analysis set.**

|  | **Baseline STL ≥60 mm** | | **Baseline STL <60 mm** | |
| --- | --- | --- | --- | --- |
| **Baseline characteristics** | **FTD/TPI plus BEV (*n* = 76)** | **FP plus IRI plus BEV (*n* = 75)** | **FTD/TPI plus BEV (*n* = 107)** | **FP plus IRI plus BEV (*n* = 109)** |
| Sex |  | | | |
| Male | 37 (48.7) | 41 (54.7) | 51 (47.7) | 50 (45.9) |
| Female | 39 (51.3) | 34 (45.3) | 56 (52.3) | 59 (54.1) |
| Age, y |  | | | |
| Median (range) | 67.5 (41-82) | 68.0 (40-82) | 67.0 (25-84) | 69.0 (32-81) |
| <65 | 25 (32.9) | 26 (34.7) | 46 (43.0) | 38 (34.9) |
| ≥65 | 51 (67.1) | 49 (65.3) | 61 (57.0) | 71 (65.1) |
| ECOG performance status | |  | | |
| 0 | 39 (51.3) | 45 (60.0) | 75 (70.1) | 67 (61.5) |
| 1 | 37 (48.7) | 30 (40.0) | 32 (29.9) | 42 (38.5) |
| *RAS* status |  | | | |
| Wild type | 30 (39.5) | 33 (44.0) | 44 (41.1) | 41 (37.6) |
| Mutant | 46 (60.5) | 42 (56.0) | 63 (58.9) | 68 (62.4) |
| Primary tumor location | |  | | |
| Left side | 57 (75.0) | 57 (76.0) | 80 (74.8) | 81 (74.3) |
| Right side | 19 (25.0) | 18 (24.0) | 27 (25.2) | 28 (25.7) |
| No. of metastatic lesions | |  | | |
| 0 or 1 | 17 (22.4) | 21 (28.0) | 50 (46.7) | 49 (45) |
| ≥2 | 59 (77.6) | 54 (72.0) | 57 (53.3) | 60 (55) |
| Time to progression in first-line treatment | |  | | |
| ≥9 mo | 44 (57.9) | 46 (61.3) | 76 (71.0) | 74 (67.9) |
| <9 mo | 32 (42.1) | 29 (38.7) | 31 (29.0) | 35 (32.1) |
| Biologics used in first-line treatment | |  | | |
| Anti-EGFR antibody | 17 (22.4) | 18 (24.0) | 18 (16.8) | 16 (14.7) |
| BEV | 59 (77.6) | 57 (76.0) | 89 (83.2) | 93 (85.3) |

Data are presented as *n* (%) or median (range).

BEV, bevacizumab; ECOG, Eastern Cooperative Oncology Group; EGFR, epidermal growth factor receptor; FP, fluoropyrimidine; FTD/TPI, trifluridine/tipiracil; IRI, irinotecan; mo, months; *RAS*, rat sarcoma virus; STL, sum of the diameter of target lesions; y, years.

**Supplementary Table 6. Subsequent post-study treatment.**

|  | **FTD/TPI plus BEV** | **FP plus IRI plus BEV** |
| --- | --- | --- |
|  | **(*n* = 197)^a^** | **(*n* = 199)^a^** |
| Third-line treatment^b^ | 118 (79.2) | 104 (78.8) |
| Same treatment as that in the protocol^b^ | 6 (4.0) | 8 (6.1) |
| Best supportive care^b^ | 25 (16.8) | 20 (15.1) |
| Third-line drug (overlapped) | **(*n* = 118)** | **(*n* = 104)** |
| IRI | 104 (88.1) | 24 (23.1) |
| FTD/TPI | 0 | 44 (42.3) |
| BEV | 47 (39.8) | 33 (31.7) |
| Anti-EGFR antibody | 6 (5.1) | 17 (16.3) |
| Regorafenib | 1 (0.8) | 12 (11.5) |

Data are presented as *n* (%).

^a^67 patients (FP plus IRI plus BEV) and 48 patients (FTD/TPI plus BEV) were under the protocol treatment as of the cutoff date.

^b^Percentages have been calculated using 149 and 132 as the denominator for the FTD/TPI plus BEV and FP plus IRI plus BEV groups, respectively.

The subsequent treatment does not include any patients who withdrew from the study.

BEV, bevacizumab; EGFR, epidermal growth factor receptor; FP, fluoropyrimidine; FTD/TPI, trifluridine/tipiracil; IRI, irinotecan.

**Supplementary Fig. 1. Randomization scheme.**


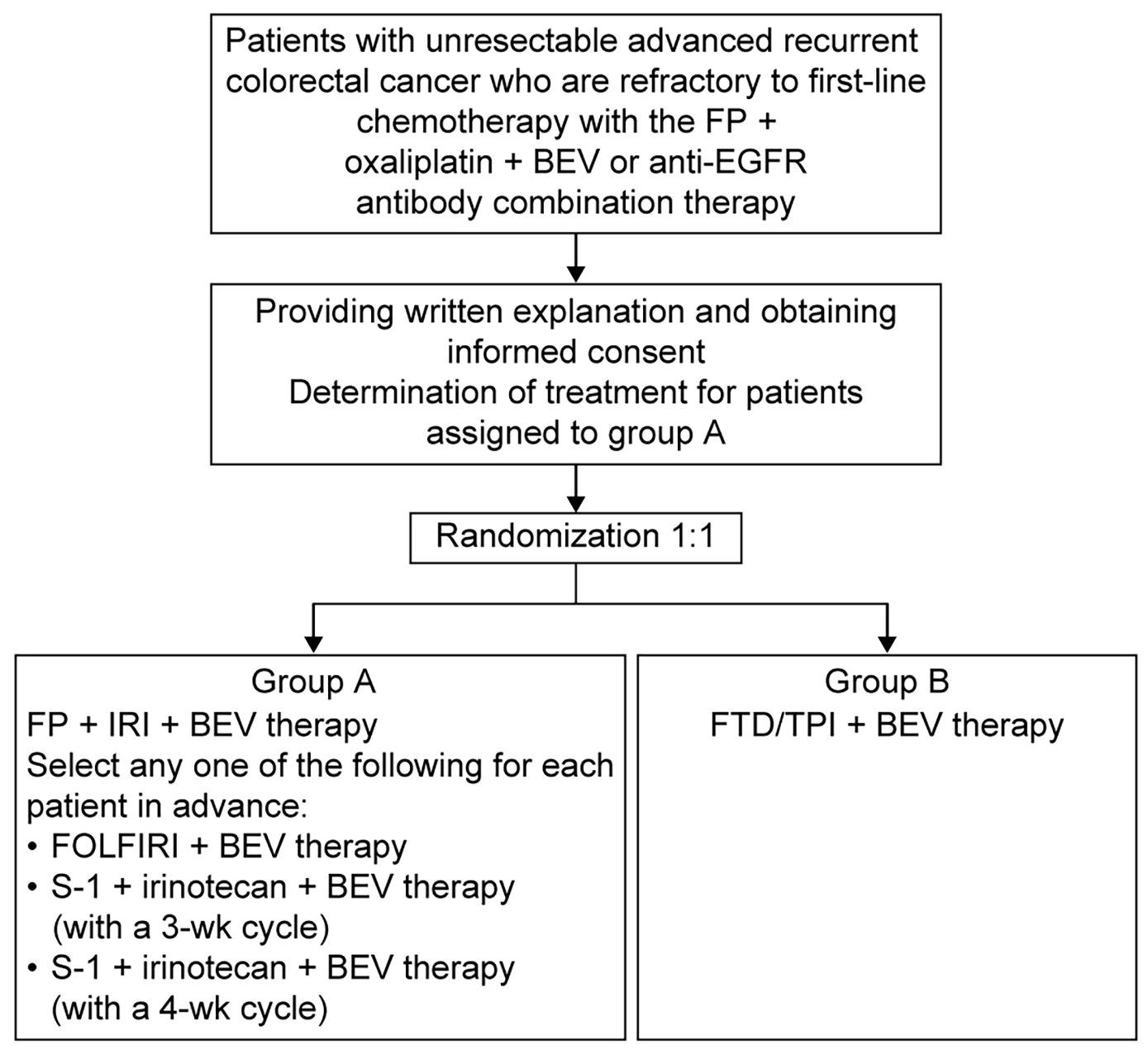


BEV, bevacizumab; EGFR, epidermal growth factor receptor; FOLFIRI, 5-fluorouracil, leucovorin, and irinotecan; FP, fluoropyrimidine; FTD/TPI, trifluridine/tipiracil; IRI, irinotecan.

**Supplementary Fig. 2. HR for overall survival by the baseline sum of the diameter of target lesions.**


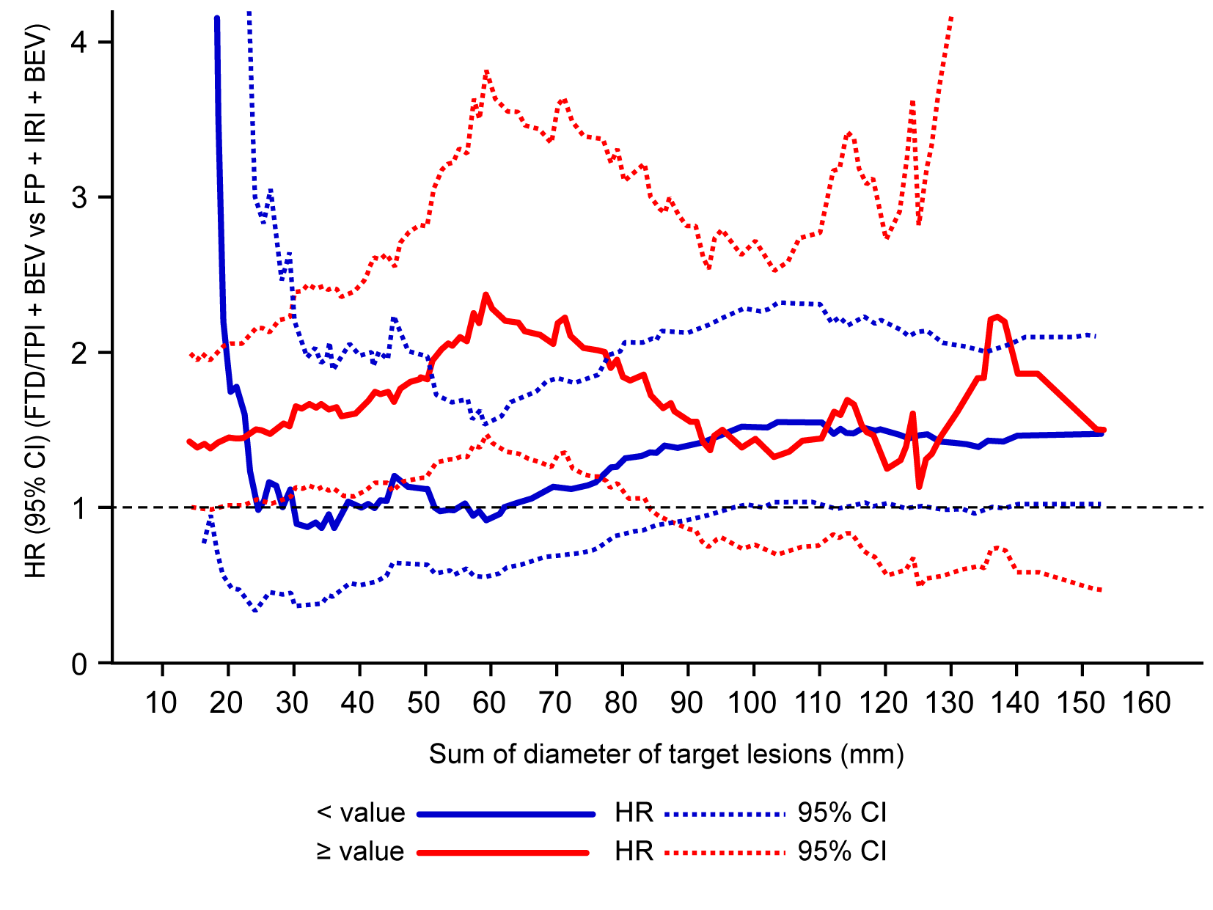


BEV, bevacizumab; CI, confidence interval; FP, fluoropyrimidine; FTD/TPI, trifluridine/tipiracil; HR, hazard ratio; IRI, irinotecan.

**Supplementary Fig. 3. Time to deterioration in QoL score: (a) EQ-5D-5L score and (b) each domain of the EORTC QLQ-C30.**


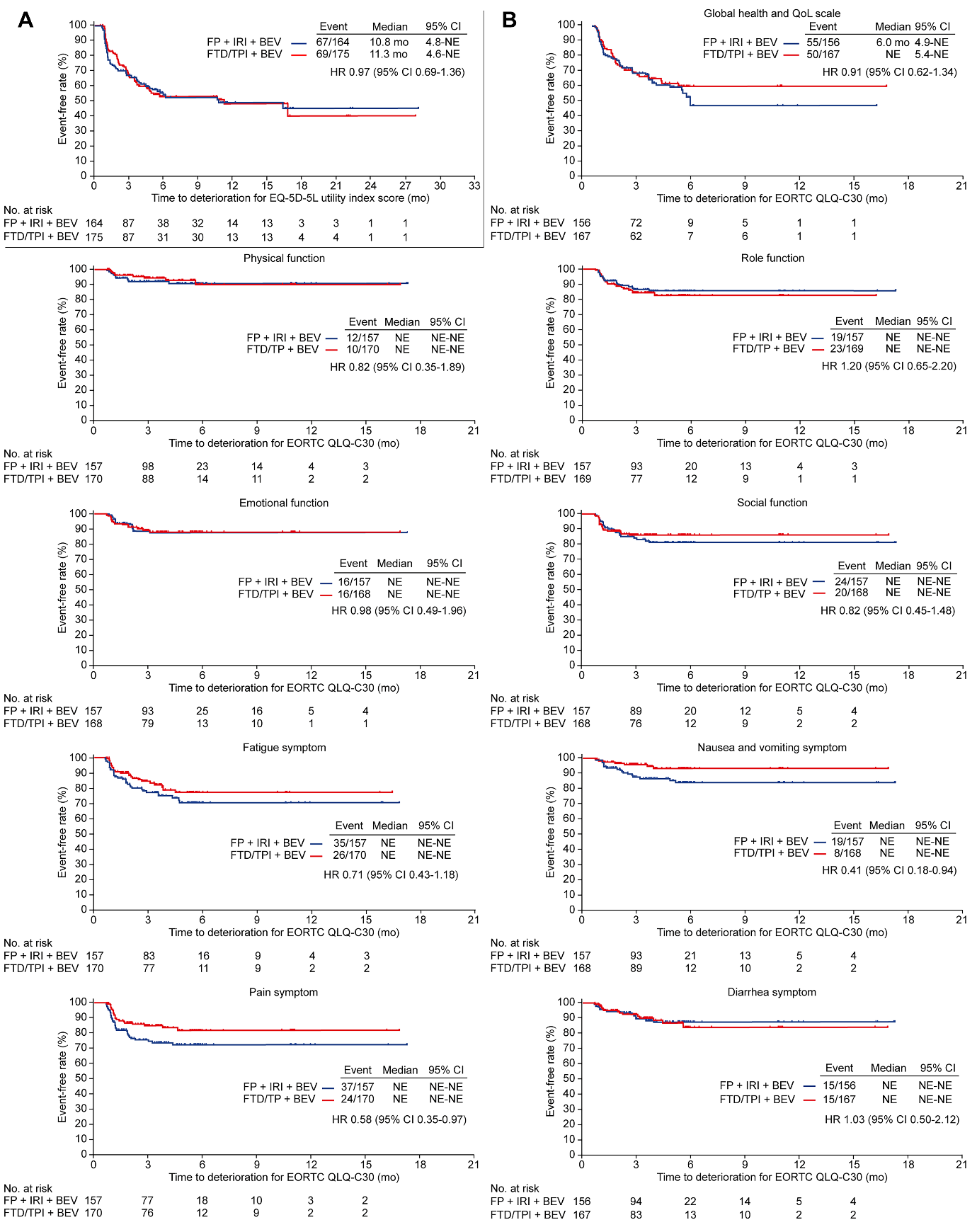
BEV, bevacizumab; CI, confidence interval; EORTC QLQ-C30, European Organisation for Research and Treatment of Cancer Quality of Life Questionnaire Core 30; EQ-5D-5L, 5-level version of EuroQoL; FP, fluoropyrimidine; FTD/TPI, trifluridine/tipiracil; HR, hazard ratio; IRI, irinotecan; mo, months; NE, not estimable; QoL, quality of life.
